# Current Perspectives on Therapy Dog Welfare in Animal-Assisted Interventions

**DOI:** 10.3390/ani7020007

**Published:** 2017-02-01

**Authors:** Lisa Maria Glenk

**Affiliations:** Comparative Medicine, The Interuniversity Messerli Research Institute of the University of Veterinary Medicine Vienna, Medical University Vienna and University of Vienna, Vienna 1210, Austria; lisa.molecular@gmail.com; Tel.: +43-677-6178-0469

**Keywords:** therapy dog, animal welfare, stress, behavior, cortisol, animal-assisted intervention, animal-assisted therapy, animal-assisted activity

## Abstract

**Simple Summary:**

In animal-assisted interventions (AAIs) animals are used as adjuncts to therapy to positively affect human health. The practice of implementing dogs into therapeutic environments is emerging and as a result, there has been a growing scientific interest on human health outcomes over the past decades. Research efforts into the canine perspective of AAIs have been scarce. Accordingly, there is little consensus on the impact of such interventions on the animals involved. This paper aimed to contribute to the limited body of knowledge by reviewing available studies on therapy dogs’ welfare during AAIs. Moreover, discussion of theoretical and methodological issues, implications for practice and suggestions for future research are provided.

**Abstract:**

Research into the effects of animal-assisted interventions (AAIs) has primarily addressed human health outcomes. In contrast, only few publications deal with the therapy dog experience of AAIs. This paper provides an overview on potential welfare threats that therapy dogs may encounter and presents the results of a review of available studies on welfare indicators for therapy dogs during AAIs. Previous investigations used physiological and behavioral welfare indicators and dog handler surveys to identify work-related stress. Research outcomes are discussed in the light of strengths and weaknesses of the methods used. Study results suggest that frequency and duration of AAI sessions, novelty of the environment, controllability, age and familiarity of recipients modulate animal welfare indicators. However, this review reveals that currently, clear conclusions on how the well-being of dogs is influenced by the performance in AAIs are lacking due to the heterogeneity of programs, recipient and session characteristics, small dog sample sizes and methodological limitations. This paper further aimed to identify unresolved difficulties in previous research to pave the way for future investigations supporting the applicability of scientific findings in practice.

## 1. Introduction

Since their domestication more than 30,000 years ago, dogs have played a significant role in the evolution of mankind [1]. During this time span, the morphologic, behavioral, and cognitive repertoire of the domestic dog has been shaped by selective breeding for specialized abilities and phenotypes, resulting in a remarkable diversity within a single species. Dogs have assisted humans with numerous tasks including protection, hunting and herding. It has been evidenced that the adaption of comparable socio-communicative abilities in dogs and humans has contributed to the development of special social skills that allow dogs to comprehend human social and communicative behavior [2]. Dogs not only perform well in reading and interpreting human gestures but seem to facilitate pro-social human behavior by increasing their owner’s social attractiveness, stimulating conversations and friendly behaviors from strangers [3,4]. Over the past decades, a growing body of research has underlined their role as social companions in human health promotion [5,6].

Animal-assisted interventions (AAIs) seek to positively affect human health by utilizing animals as adjuncts to therapy. The steadily increasing popularity of these programs is supported by the fact that they deliver health benefits that are primarily associated with pet ownership also to non-pet owners [7]. Most commonly, AAI programs are designed to facilitate therapeutic progress or simply to enhance the quality of life of patients or residents. AAIs are broadly defined as any practice that involves animals as a part of a therapeutic or ameliorative process [8]. Further specification in regard to terminology differentiates between animal-assisted therapy (AAT) and animal-assisted activity (AAA). Basically, programs of the AAT-category are carried out by professionals involved in preventive, curative, promotional or rehabilitative health care services, offering goal-directed procedures with animals as an integral part that require documentation and evaluation of intervention progress and outcomes. In contrast, AAAs are delivered spontaneously, may be carried out by professionals or volunteers and lack a previously defined goal, documentation and evaluation [8]. The feasibility of this classification has been challenged by Palley et al. [9] because terminology in the literature is not standardized, resulting in a broad use of AAT also for programs that per definition do not fit into the category.

The high prevalence of dogs in therapeutic environments has been linked to their availability, trainability and consequently predictability. In AAIs, contact is usually initiated by the so-called therapy dog while intensity and duration of the procedure vary with the recipient’s particular situation-specific needs, addressing both the physical and emotional components of health [10]. It has been claimed that dogs can enhance motivation to participate in therapeutic activities via intrinsic activation [11]. In addition, the presence of a dog has been linked to increased stress resistance via social support, evidenced by lower cortisol levels during venipuncture or situations of social evaluative threat [12,13]. Moreover, dog companionship seems effective in modulating subjective pain as self-reported pain levels post-surgery were lower if patients interacted with a therapy dog [14,15]. Recently, it has been demonstrated that interaction with a friendly dog can increase the short-term tolerance of aversive acoustic stimulation [16]. Psychological benefits of human-animal interaction include decreased levels of anxiety, sadness, loneliness and increased social functioning and secretion of the neuropeptide oxytocin during human-animal contact has been suggested to be a modulating factor [17].

### 1.1. Do AAIs Raise Concerns for Animal Welfare?

Does the integration of dogs in AAIs promote human well-being at the expense of animal well-being? Quality perceptions in AAIs are high and these practices have been considered not to be particularly stressful for the participating dogs [18]. Although reactions toward therapy dogs in institutions are merely positive, adverse interactions have been reported. Inappropriate behaviors including teasing and mistreatment of visiting dogs were observed in AAI recipients and staff members [19,20,21]. A study on the effectiveness of AAT on children with multiple disabilities had to be terminated early because of the deteriorating health of the therapy dog, preceded by symptoms of stress like excessive panting and tiredness [19]. The current animal welfare debate has demonstrated relatively broad agreement that AAIs may raise welfare concerns for participating animals. As a result, extended guidelines to reduce work-related strain and to increase the quality of life in therapy animals have been published by the International Association of Human-Animal Interaction Organizations, IAHAIO [22]. In 2016, a European Union-funded project on therapy dog training involving partners from Germany, Norway and Poland led to publication of a handbook on basic requirements and knowledge for dogs in AAAs [23]. In 2015, an initiative by the Austrian Ministry of Labor, Social Affairs and Consumer Protection resulted in the legal regulation of therapy dog certification, including the evaluation of the human-dog team and regular health-, temperament- and behavior check-ups to re-evaluate an animal’s disposition [24]. However, precise criteria necessary to ensure welfare in therapy animals are still missing due the diverse spectrum of types of AAIs and as a result, a universal standardization of guidelines remains challenging [25].

### 1.2. Ethical Considerations

The moral legitimacy of integrating animals in AAIs has been challenged by Zamir [26], who proposed six violations of moral status including limitations of freedom and life determination, as well as training, social disconnection, injury, and instrumentalization. Taken these objections into account, the moral justification of pet ownership per se necessitates reconsideration. Under human management, only few, if any animals may freely engage in natural behavior. In contrast, if animals can actually benefit from the interactions with humans, their involvement as therapeutic aids in AAIs may be ethically justifiable. If participation in AAIs supports some of the animal’s own interests, human interests could be easily met without exploiting the animal. According to Zamir [26], this refers primarily to dogs and horses and excludes rodents, birds and wild animals.

Iannuzzi and Rowan [27], conducted a survey using questionnaires and phone interviews to identify practices of AAIs that may raise ethical concerns for animals. According to the respondents, resident animals especially should be closely monitored for signs of fatigue and given ample opportunities to rest. In visitation programs, limited access to water and high room temperatures in institutions were identified as a serious problem. Moreover, survey respondents claimed that the duration and number of visits should be limited to 60 min and 3 sessions per week, respectively.

### 1.3. Selection Protocols and Certification

To become a registered AAI-team, expert evaluation, training and successful certification require both personal and financial commitment on the part of the animal handler. Therapy dogs are usually required to complete special training and a temperament test to meet the criteria established by the institutions that certify animal handlers and dogs. Among these conditions is the ability to cope with strange situations, to remain calm and confident under stressful situations and to be reliable with visual or vocal commands [28,29,30]. Although certification protocols vary significantly between organizations, minimum common prerequisites include immaculate health status, the willingness to interact with unfamiliar people and the absence of behaviors that could jeopardize AAI recipients [25,31]. Unfavorable behaviors include aggression, jumping up or on, mouthing, biting, and dodging [31].

Although many dog owners would consider their animals as ideal companions, this does not necessarily make them good candidates for AAIs. Some AAI programs involve shelter dogs with the aim of enhancing socialization and adoption rates [20,32]. However, compared to privately-owned pet dogs, evaluation of a shelter animal’s disposition for AAIs may be even more challenging because of lacking information on individual history, previous experiences and preferences [20].

Experimental evaluation of both veterinarian expert and handler perceptions of dogs’ suitability to work in AAIs, comparing therapy dogs and pet dogs was conducted by Mongillo et al. [31]. Handler self-reports and veterinary expert opinions categorized therapy dogs as suitable, suitable with reserve and unsuitable for AAIs. Behavior screening criteria were controllability, reliability, predictability, social and problematic behavior (i.e., aggressiveness, fear, interaction avoidance). In addition, the dogs were also subjected to a simulated AAI-session in which they were confronted with challenging behaviors from an actor patient and caregiver. The provocative actions included unusual movements, noise, hugging the dog, patting the dog on the head, seizing the dog’s harness and distraction of the session. Unexpectedly, no differences in the display of stress behaviors emerged between the dog groups, suggesting that therapy dogs were not able to deal more efficiently with AAI-related challenges than ordinary family dogs. Study outcomes also revealed that dogs showing aggressiveness and fear to a certain degree may still be judged suitable with reserve by their handlers. The roleplay scenario was considered appropriate for the identification of dogs that fully meet the AAI-suitable criteria.

### 1.4. Tactile Contact

Previous research has demonstrated that positive interactions between humans and dogs can stimulate individual and mutual positive effects, confirmed by hormonal changes that emerge in both humans and dogs [33,34]. In shelter dogs, as little as 15 min of gentle stroking is sufficient to deliver a calming effect by means of lower cortisol levels and behavioral changes [35]. During interspecies play sessions, working dogs that experienced affiliative interactions including tactile contact and verbal praise had lower cortisol concentrations than dogs that were engaged in authoritarian play based on exercises and commands [36].

However, there is evidence that not all kinds of physical interaction that humans enjoy (e.g., petting the head, face to face contact, hugging, kissing) are equally perceived as pleasant by dogs. For example, children who want to engage in close physical contact with either a familiar or unfamiliar pet dog may provoke situations of serious conflict [37]. Therapy dogs are expected to tolerate physical intimacy with strangers and to remain there calmly for minutes. Given the fact that dogs have been primarily bred for assisting humans in hunting, herding and guarding, they were supposed to recognize family members and be suspicious of unfamiliar individuals and/or intruders [38]. Accordingly, being approached, petted and hugged by strangers in unfamiliar environments, which is commonly featured in AAIs, may elicit discomfort in dogs [30].

Kuhne et al. [39] measured dogs’ behavioral responses to human tactile contact. In their laboratory studies, dogs displayed increased gestures of appeasement, redirected behaviors and displacement activities in response to physical restraint or if they were petted at the head, muzzle, paw or tail, suggesting that these actions caused mild levels of discomfort. No increase of such behaviors was found if dogs were petted at the chest and neck. The frequency of behaviors also varied depending on whether dogs were handled by a familiar or unfamiliar person [40]. More recently, Kuhne et al. [41] replicated their experiments including the assessment of autonomic arousal via heart rate variability measurements. Increased parasympathetic activity and lower heart rate were registered if dogs were petted at the tail base, shoulder, or chest, indicating relaxation. Appeasement gestures including paw lifting, looking or moving away and lip licking correlated positively with heart rate and negatively with vagal tone, suggesting higher sympathetic nerve stimulation.

### 1.5. Environmental Factors

The demands therapy dogs encounter during their performance in therapeutic environments go beyond the challenge of accepting close social contact with strangers. For example, research has demonstrated that walking on different floor substrates (e.g., parquet, plastic, stairs, iron grids) can trigger physiological arousal also in dogs with no previous history of floor fearfulness [42]. Therapy dogs are expected to remain comfortable with a variety of potentially challenging stimuli including wheelchairs, crutches, metal walkers, sudden noise, white coats, high temperatures in their working environments [27,30,31].

In their literature review on the efficacy and safety of dog-assisted interventions with regard to transmission of zoonotic diseases in hospital settings, Bert et al. [43] suggest that the benefits of visiting dogs outweigh the risks, which were considered to be minimal. However, AAI recipients colonized with infectious pathogens pose a risk of transmitting hospital-acquired diseases also for dogs and their handlers [44]. Thus, proper veterinary screening and communication with animal handlers who are responsible to provide the best practice for preventative health to their therapy dog are mandatory [25].

### 1.6. Animal Welfare Assessment

In 1986, Broom introduced a widely cited definition of the animal welfare concept by referring to an animal’s attempts to cope with the environment [45]. Similarly, the welfare state of an individual relates to its abilities to deal with the stress imposed on it by the environment [46]. Moreover, animal welfare is strongly affected by predictability and controllability of the environment [47]. When studying animal welfare, measurements should reliably reflect and translate the actual state (i.e., physical, emotional) of an individual into an objective description that can be delivered and agreed upon by different observers. Additionally, statements about animal welfare are usually based on measurements that include physiological and/or behavioral parameters [48]. Clinical health and the absence of pain do not necessarily indicate good welfare because the freedom from fear and distress and the freedom to express natural behavior are crucial [49]. Research parameters are either indicators of physical health or measures that are indicative of stress due to its pivotal role for well-being and the development and/or progression of diseases. Chronic stress is commonly preceded by subsequent and prolonged exposure to stressful conditions. Hence, in welfare research, it is essential to capture short-time effects and changes that, in sum and/or over time may pose a severe challenge to animal’s health [48]. Findings from a recent study in a veterinary clinic indicate that dogs rated as more stressed by their owners and veterinarian experts engaged less in social contact with an unfamiliar person [50]. Accordingly, stress levels in dogs may not only impact animal welfare but may have broad-ranging effects for the quality of inter-species social interaction.

According to researchers’ perceptions, in-depth investigation into the animal experience of AAIs is required [5]. Preliminary research into therapy dogs’ welfare has been complicated by the diversity of programs and heterogeneity of study protocols [51]. Thus, the aim of this paper is to evaluate effects of human-animal interaction during AAIs on participating dogs by elucidating working conditions including environment, recipients and session characteristics as well as dog welfare indicators.

## 2. Literature Review

Inclusion criterion for reviewed literature was the publication in a peer-reviewed scientific journal or as a Master´s or PhD thesis, including case reports and original research. Key word search terms were: therapy dog, animal welfare, stress, arousal, behavior, AAI, AAT, AAA. The literature search resulted in nine relevant papers, of which two were case reports and one a master thesis.

As demonstrated in Table 1, welfare assessment in therapy dogs has been carried out at multiple therapy sites including in-patient and out-patient facilities, schools and university [28,52,53,54,55,56,57,58,59,60]. All studies provided declarations whether the dogs performed AAA or AAT or both. The majority of studies investigated visitation programs with adult recipients arranged in group settings.

Table 2 provides an overview on session characteristics and significant findings, indicating that the way AAIs were conducted varies considerably across studies [28,52,53,54,55,56,57,58,59,60]. Session durations differed markedly from 10 min to 8 h and there was also a great variety in between-session intervals, with some dogs participating in AAIs on a daily basis, others several times a week, weekly, or less. Dog welfare indicators were cortisol (assessed in saliva, feces and hair), stress-related behaviors, clinical parameters, and animal handler perceptions. Stress-related behaviors included lip licking, panting, paw lifting, trembling, body shaking, vocalizing, withdrawal and self-grooming. Behavior was accessed via direct [28,55] and video-taped [28,57,58,59,60] observations of the dogs’ responses or according to handler reports [54]. Intra- and/or inter-observer reliability were reported in three studies and ranged from 90% to 93% [58,59,60]. Behavioral sampling was carried out across the duration of the whole session [28,57,58,60], during one minute after a two-hour work shift [55] or during sequences where the dog was petted for five minutes [59]. Common AAI interaction schedules included verbal praise, petting, gentle scratching, brushing the dog’s fur, walking the dog on- or off-lead, obedience commands, throwing or hiding dog toys, mild exercises. Only one study reported whether other therapy dogs were enrolled simultaneously in the experimental sessions, indicating that up to eight handler-dog teams shared a big room but worked on separate spaces to avoid interaction between the dogs [59].

### 2.1. Case Reports

Assessing clinical indicators, behavior and cortisol levels in a shelter dog over the course of 6 months, Piva et al. [28] determined how these variables were affected by the procedure of adoption and integration as a resident dog. After being rehomed in a nursing home for the elderly, a dog was regularly enrolled in AAA group sessions. The dog had a previous history of stereotypic autogrooming, causing her to have an acral lick granuloma, which gradually disappeared over the course of the study. Evaluation of the dog across three time points during the AAA program indicated that over time, the dog appeared to be healthy, playful, engaged in social interaction and exploration. Hair cortisol and stress symptoms including tachycardia, tachypnea, nose and lip licking, hypervigilance and walking-pacing tended to decrease progressively over time, suggesting that the dog was positively integrated in the new environment. In the case report by Palestrini et al. [60], a therapy dog was enrolled in 20 AAT sessions in a pediatric hospital and sessions were carried out during post-operative awakening, 2 h after surgery. Stress level evaluation was carried out via heart rate recordings and analyses of the stress-related behavior, exploration, passive behavior, environmental orientation and interaction with children, animal handler and other people (i.e., staff, parents). Heart rates remained within the physiological range and did not differ whether or not children interacted with the dog during the sessions. Moreover, the dog never tried to withdraw from the intervention and other behavioral variables did not vary across the sessions. High levels of panting were attributed to the relatively high room temperature. The authors concluded that participation in the program did not result in any welfare concerns for the dog [28,60].

### 2.2. Master's Thesis

An assessment of fatigue in carefully selected therapy dogs by measuring the dogs’ motivation in a behavioral task was carried out by Barstad [57]. Dogs were subjected to a cognitive task in which they were confronted with a treat in a closed bucket on a resting day and pre-post intervention. Performance across the behavioral tasks did not reveal any signs of fatigue, tiredness or lack of motivation as a consequence of AAI-related activities. Behavior and interaction patterns between the dog, its handler and recipients were video-analyzed during session 2 and session 10 across a 12-week intervention program. Study outcomes suggest that in the later session, dogs were less focused on their handlers but more responsive to commands. No differences in stress or displacement behaviors between session 2 and session 10 were found. However, few incidents of rough handling and withdrawal from the recipient were reported. In these instances, handlers intervened immediately by explaining to the recipients how to interact appropriately with the dog. The author concludes that therapy dogs’ behavior during AAIs and their performances in a cognitive task are constant over time. Minor symptoms of stress were observed in the animals, suggesting that supervision and instruction of recipients are needed but the benefits AAIs deliver to human health outweigh the costs [57].

### 2.3. Original Research

The studies by Haubenhofer and Kirchengast [52,53] and Marinelli et al. [54], exhibit a high variability of AAI settings with regard to the therapeutic environment, the number and age of recipients and session arrangements. Haubenhofer and Kirchengast reported that dogs’ salivary cortisol concentrations were higher on days with AAIs if compared to a resting day [52,53]. In addition, cortisol levels varied with the duration of sessions and the number of visits per week. Owner surveys revealed that sessions between 1 and 3 h were perceived to be more intense, with fewer breaks scheduled than longer performances. Emotion attribution according to handlers indicated that they perceived their dogs to be more likely physically strained from therapeutic work than themselves. The authors suggest that therapy dogs should be given several days of rest after participation in an AAI to reduce over-arousal [52,53]. The finding of higher cortisol levels associated with AAIs was corroborated by King et al. [55] who found an increase in therapy dogs’ salivary cortisol after 60 min of performed AAT in hospital. Stress behaviors sampled during one minute after two hours of AAT included panting, pupillary dilation, yawning, whining, and air licking. Behavior did not differ if dogs were subjected to two minutes of a quiet time-out after 60 min. However, increases in salivary cortisol levels correlated with the occurrence of stress behaviors. Less behavioral signs of stress were observed if dogs had two years of experience in AAT or more and/or were older than 6 years [55]. Marinelli et al. [54] assessed working conditions and handler reports on stress-related behaviors in dogs involved in AAA and AAT over a period of three years. An increase in both the frequency of sessions and the number of participants was registered for each dog with an overall lower perception of the quality of the intervention according to dog handlers. Recipient age was related to the expression of stress-related behaviors, which were more prevalent in sessions with children under 12 years. Environmental conditions identified inadequate for dog welfare were interferences, high temperatures and lack of space [54].

In contrast to the results by Haubenhofer and Kirchengast [52,53] and King et al. [55], no differences in salivary cortisol concentrations comparing working days with AAIs and resting days at home were found by Glenk et al. [56,58], and Ng et al. [59]. Two studies were conducted by Glenk et al. to investigate the effects of AAIs in in-patient facilities with adult recipients on participating dogs by analyzing salivary cortisol and behavior. In their first study therapy dogs were enrolled in group sessions in psychiatric treatment and salivary cortisol concentrations were found to be significantly lower in experienced dogs that were kept off-lead and allowed to roam freely during the sessions. No differences were found between pre-post session levels in experienced dogs on-lead and therapy dogs in training [56]. In a follow-up study with recipients undergoing substance abuse treatment, no significant changes in behavior were registered over a period of five weeks, however decreases in salivary cortisol were significant in the last two sessions [58]. The authors concluded that no signs of acute stress were detectable in the dogs across the two studies and that therapy dogs may benefit from increased controllability if they can roam freely and interaction with a group of increasingly familiar recipients [58]. Ng et al. [59] investigated how therapy dogs are affected by the environment, comparing sequences where the dogs were at home with their handlers, participated in an AAA with university students or were introduced to a novel environment where they quietly sat with their handlers. A significant increase in salivary cortisol concentrations was found only in the novel environment compared to the home or AAA setting. No differences in the display of stress-related behaviors emerged between the three conditions. Behavioral differences were only found for postural state, resulting in more standing and ambulating if stimulated by interaction with strangers during the activity setting. The authors speculated that rises in cortisol levels may not necessarily be accompanied by behavioral indicators of stress as has been previously suggested by Handlin et al. [61].

In another ongoing study, therapy dogs’ behavior and salivary cortisol levels during AAIs in pediatric oncology are complemented by demographic variables and handler reports of their dogs’ temperament, behavior and the course of action during AAI sessions [62].

## 3. Discussion

In AAI research, qualitative (i.e., subjective evaluation of either a professional or caretaker) and quantitative (i.e., objective measures of the immediate response such as behavior and physiological parameters) methods or a combination of both are used [63]. The current body of research has assessed dog welfare in AAIs via animal-orientated assessment of behavioral and physiological measures or questionnaires and interviews that rely on human interpretation of animal well-being. Cortisol and behavior were the most commonly used welfare indicators. The study of bodily and behavioral responses may provide a more objective reflection of the animal’s perception than subjective attributions from a human observer. However, evaluation of physiological and behavioral parameters and putting them into context also require a human interpreter. Accordingly, any methodology that builds on human judgment is susceptible to individual interpretation [20]. It must further be acknowledged that endocrine, autonomic and immune markers that respond to stress are not solely stress-associated systems but do serve other functions in homeostatic maintenance [64]. Precaution needs to be undertaken when interpreting behavioral and physiological data in the context of animal welfare as variations in individual responsiveness have to be considered [65].

### 3.1. Physiological Responses

Studies on therapy dog welfare have analyzed salivary cortisol as a reference biomarker for physiological arousal. An activation of the hypothalamic-pituitary-adrenal (HPA)-axis in response to stress and fear results in increased secretion of the adrenocortical hormone cortisol, which can be determined not only in plasma but also non-invasively in saliva [66], hair [67] or feces [68]. Saliva collection was the most common method for cortisol determination across the reviewed studies as it can be carried out relatively easily also under non-laboratory conditions such as in AAI-environments. Dog handlers or owners can be instructed and trained to collect their dogs’ saliva so that sampling procedures do not cause any additional stress.

Both short-term increases and decreases in salivary cortisol were described in therapy dogs, suggesting that participation in AAI programs may have both stimulating and calming effects. However, the current body of knowledge does not provide any clear conclusions whether these variations can be attributed to certain intervention practices or content. Dogs that did not show differences in cortisol secretion on working or resting days may be well-adapted to their AAI working routines so that these practices are probably not straining for them. Further, it has been speculated that cortisol levels may decrease throughout the intervention when dogs are given the opportunity to withdraw or move freely and thus, are able to choose whether or not to engage in close contact with recipients [56,58].

Recent research has shown that salivary cortisol in healthy dogs is characterized by a high intra- and interspecific variability and modulated by a variety of demographic and environmental factors, limiting the generalizability of studies that used it as sole marker [66,69]. Caution is further advised in the interpretation of salivary cortisol results as a biomarker of welfare evaluation because increased levels may also reflect positive arousal and excitement [59]. Moreover, it must be recognized that both positive affective state and moderate arousal are likely to maximize performance and desired behaviors in dogs [70]. While it is generally agreed that short-term variations in cortisol are necessary for effective stress coping, excessively high levels for prolonged periods of time negatively affect health and dampen immune function [71].

Patterns of cortisol responses are complex and individually variable in magnitude, duration and speed of change. Salivary cortisol levels reflect plasma cortisol concentrations with a delay of 20–30 min [72]. Interpretation of salivary cortisol results can be challenging if group differences between basal and later concentrations, peaks and the time needed to return to initial levels limit the informative value. Thus, if samples are collected at previously defined time points, the intervention effect may not be effectively represented in all animals. However, in understanding HPA-related responses to stress it is crucial to distinguish between animal-specific differences and intervention effects. To yield reliable results, study subjects should be in a good overall health and any medication intake that could affect systemic cortisol levels has to be sufficiently considered.

Interpretation of cortisol responses is further complicated by its differential function in acute and chronic stress conditions. This dichotomy of has been well described in shelter dogs. If newly placed into a shelter, dogs experience an increase in cortisol levels. However, if they stay at a shelter for longer periods of time, their salivary cortisol concentrations appear to be significantly lower than from conspecifics kept as pets or in working kennel facilities [66]. To date it is not fully understood whether the adaption in cortisol is caused by dysregulated HPA-axis function or habituation to the shelter environment [73] but with regard to welfare assessment in the AAI context, salivary cortisol may not be a suitable marker to investigate the intervention effect in shelter dogs.

If combined with behavioral and other physiological measurements, the study of salivary cortisol responses is likely to yield more reliable results in reflecting animal welfare. For example, in dogs, decreases in secretary immunoglobulin A (IgA) have been linked to stressful experiences and negative correlations with cortisol were reported [74,75,76]. A stress-induced discharge of the sympathetic nervous system stimulates secretion of noradrenaline and adrenaline, accelerating heart rate [77]. Only one of the reviewed studies used heart rate as a physiological measure [60] although assessments of heart rate and heart rate variability have previously been applied in dog welfare science [41,78,79]. Investigating the effects of human-horse interaction on autonomic regulation in horses involved in AAA, Gehrke et al. analyzed heart rate variability in 24-h heart rate recordings [80]. Clearly, further investigation into therapy dogs’ physiological measures is needed.

### 3.2. Behavior

Observation and interpretation of dog body language and posture have been widely used to analyze human-animal relationships. Perhaps no aspect of the dog’s behavioral repertoire elicits more controversy than stress-related behaviors or the so-called calming signals, appeasement or displacement gestures that communicate mild states of discomfort and uncertainty [81]. Although it has been widely accepted that these subtle cues (e.g., yawning, lip licking, body shake, paw lifting, panting, tail wagging) are substrates of intra- and interspecies communication, one of the most recently debated issues is whether these behaviors actually represent stressful conditions in dogs.

Behavioral concomitants of stress-related physiological conditions can be commonly observed and have been previously described in dogs after exposure to noise and fearful stimuli [42,82]. In response to spatial restriction, dogs showed enhanced locomotion, yawning, paw-lifting and body shaking [83]. In addition, lip licking and yawning preceded situations of social conflict [84] and paw-lifting was associated with a state of conflict, confusion and fear of punishment [85]. In guide dogs, lip licking and panting during were more likely displayed by dogs that performed poorly on a task [86]. On the other hand, lip licking and body shaking were attributed to increased positive arousal and affiliative social interaction [87,88].

No significant factors of human-animal interaction on dogs’ expression of stress-related behaviors were found across the reviewed studies except for age, where Marinelli et al. reported more stress behaviors if dogs interacted with children younger than 12 years [54]. Consistently high durations of panting across multiple sessions were linked to high temperatures in a pediatric ward during post-operative awakening [60]. The absent effect of a quiet play session with the owner between AAIs may be due to the short duration (i.e., two minutes) of the time-out [55]. Dogs subjected to a cognitive task performed equally well regardless of whether they were enrolled in AAA and AAT prior to the experiment, indicating no signs of fatigue or exhaustion [57]. In the majority of studies, lip licking was displayed more frequently than yawning, body shaking or paw lifting [57,58,59,60]. Incidences where dogs withdrew from a recipient or avoided interaction were absent or very rare [57,60].

To ensure the integrity of behavioral observation, ratings either from one or more observers need to be consistently reliable over time. Accounting for examiner judgment bias, behavioral studies should provide calculations of intra- and inter-observer reliability agreement rates that ought to be in acceptable ranges [89]. Intra-observer reliability represents the rating consistency of a single observer, comparing the same observer’s report from a recording of the same sequence viewed on two or more separate occasions. Inter-observer reliability refers to the level of agreement in two or more observers evaluating the same situation and recording the same behaviors [89]. This review found that only three out of seven studies provided intra- and/or inter-observer reliability. Besides, the current body of research does not provide sufficient evidence as to whether observation of the full duration of an intervention yields more reliable results on stress behaviors than analysis of excerpts of a session.

### 3.3. Handler-Dog Interaction and Environmental Factors

An emerging body of research has been devoted to understanding the functional properties of attachment in the human-canine bond [90]. The evolutionary base for this bond has been related to psychological and physiological processes [91]. Similar to the consistently close relationship between a human mother and her infant, a dog is securely attached to its owner if that person can serve as a secure base for reassurance during exploration of the environment [92]. If confronted with a stressful or dangerous situation, the owner represents a haven of safety for the securely attached dog to regulate its arousal by transmitting feelings of security [92]. In AAIs, animal handlers are required to recognize discomfort in their dogs and to intervene in early stages of negative arousal. However, stress perception may vary across handlers. In a survey on pet dog owners, individuals with a higher educational level were more successful in giving a correct interpretation of stress. Additionally, dog owners who failed to identify subtle behaviors of stress reported that their dogs were less stressed in comparison to respondents who were aware of the subtler signs of unease and therefore considered their own dog as highly or moderately stressed [93]. Comparably, semi-structured interviews with 10 volunteers participating in AAA with shelter dogs revealed that the familiarity with dogs’ behavioral cues, especially referring to subtle signs of discomfort, varies substantially between animal handlers [20].

Also, the suitability of an animal may vary across its life span. According to animal handler perceptions, older dogs may be more suitable because of their milder temperament [20]. Indeed, study outcomes suggest that older and experienced therapy dogs may exhibit less stress behaviors and arousal [55,58]. On the other hand, it has been proposed that aged dogs may not be able to cope as efficiently with mild stress during experimental separation from the owner than their younger conspecifics [94]. Although it has been proposed that working schedules of older dogs should be adjusted to their needs associated with cognitive and physical impairment [30], there are no investigations on retirement procedures in therapy dogs available. Similarly, throughout the literature, the number of studied subjects (*N* ≤ 21) was too small to assess potential effects of different breeds on therapy dog performance.

To date, it also remains unclear how therapy dogs are affected by the presence of conspecifics participating in the same session. Although the results by Ng et al. [59] do not point at any negative arousal associated with multiple dog sessions, it would be interesting to investigate whether dogs’ responses vary if other dogs participate in the same session and whether there is a tolerance limit (e.g., minimum of individual space required, maximum number of conspecifics nearby) to remain comfortable.

It has been proposed that anticipation and transportation to the setting may result in a triggered state of excitement but previous studies did not control for whether this effect is manifest in physiological or behavioral variables [58,59]. Likewise, it has been suggested that especially in research settings, therapy dogs should be given ample time to adapt to the new environment after arrival at the destination facility so that any arousal induced by transportation can settle [58].

### 3.4. Limitations

Throughout the past decades, science in the field of AAIs has been criticized due to the lack of controlled experiments and well-designed research methodology with a resulting predominance of descriptive or hypothesis-generating studies [95,96]. Similarly, also the generalizability of the current body of research on therapy dog welfare is limited by the number of studies, small dog sample size within studies, heterogeneity of AAI programs and the lack of suitable control groups and randomization. To date, it has not been fully understood whether positive effects on human health can be attributed to interaction with a therapy dog acting as the active ingredient, the handler who provides attention to the recipient or a combination of both [97]. In addition, it has been proposed that researcher attitudes toward the efficacy of AAIs may shape their interpretation of study outcomes and consequently, favorable results may be more likely to be reported [98]. Besides, a researcher bias may be considered if scientists are convinced of the positive effects of AAIs, with the result that they may be less likely to report unfavorable findings regarding therapy animals. Review of the AAI literature suggests a substantial influence of third party interests (e.g., humane societies, non-profit organizations and the pet-food industry) on the communication of scientific findings, especially the media coverage [95].

### 3.5. Implications for Practice

Training methods should be based on reward or positive reinforcement, which has been previously described suitable to promote affiliative human-dog relationships and increased obedience [99]. Inappropriate training methods and practices involving forced positions in which animals cannot avoid invasive social intrusions and do not have the opportunity to withdraw should be avoided. Caution is mandatory especially for therapy dogs that were trained to remain calm in response to stressful stimuli as they may not exhibit stress-associated behaviors although physiologically aroused [59]. For both visiting and resident dogs in specialized facilities, room temperatures should be moderate and ad libitum access to water and a quiet place to rest should be provided. Novel environments are exciting also for therapy dogs. Thus, they should be given ample opportunity to explore unfamiliar surroundings before they are introduced to recipients. Dogs working under familiar conditions may be less distracted and more obedient [57]. Likewise, staff members and recipients should be carefully instructed how to interact appropriately with the dog before actual contact is initiated. Certain types and intensities of interaction may be overwhelming and animal handlers should be aware of both subtle and individual behavioral cues in their dogs. Random behavior sampling and evaluation protocols may be useful in both assessing a dog’s disposition for (re-)certification procedures and to identify work-related challenges. Economic interests may be involved in the procedures of therapy dog selection, education and evaluation. As pointed out by Ng et al. [25], a conflict of interest may arise if financial gain outweighs the welfare status of the animal.

## 4. Conclusions

Overall, the current body of evidence does not raise acute concern for the emerging practice of implementing dogs into therapeutic environments. Although studies reported behavioral signs of distress and increases in cortisol levels during AAIs, none of the authors, based on their findings, suggest prohibition of these practices because of severe animal welfare constraints. A limitation of previous studies is the lack of information on causal effects and the circumstances under which the observed dog welfare indicators vary during AAIs. For example, it would be particularly interesting to investigate whether changes in dog behavior and physiology occur in response to handler or recipient action or whether they might be modulated by other factors. Thus, more detailed study into therapy dogs’ immediate responses to human action would be needed.

An enhanced understanding of how therapy dogs are influenced by environmental factors, AAI program features and human handling may foster the promotion of successful human-dog teams. Accordingly, further exploration of the canine experience of AAIs through the use of validated scientific tools is warranted. Implementation of rigorous research into both the human and therapy animal perception and their consequences for animal welfare is needed. It would be especially interesting to assess personality traits and stress levels also in animal handlers. Welfare assessment in therapy dogs brought for visitation may require different protocols than in resident animals. Innovative methods like neuroimaging and eye-tracking that have primarily been used in human research are becoming increasingly available also for veterinary science, opening new perspectives. Since only few previous studies provided clear documentation on therapeutic content, future research should focus on using/providing standardized intervention. Moreover, previous studies have predominately focused on short-term effects of participation in AAIs. Thus, investigations into how therapy dogs deal with the demands of their working schedules on a long-term basis are warranted. Shifting away from the stress induction focus toward investigation of positive welfare indicators would provide essential insight as to if and how therapy dogs can actually benefit from interaction in AAIs.

## Figures and Tables

**Table 1:** Overview on program definitions, therapeutic environment, recipients, sample of dogs and welfare indicators. AAA: animal-assisted activity; AAI: animal-assisted interventions; AAT: animal-assisted therapy.

References	AAI Type	Program Type	Environment	Recipients	Dogs (*N*)	Welfare Indicators
Haubenhofer and Kirchengast [52,53]	AAA, AAT	Visitation	Hospitals, schools, rehabilitation centers, nursing homes	Adults, children	18	Salivary cortisol, emotions according to handler
Piva et al. [28]	AAA	Resident	Nursing home	Adults	1	Clinical protocol, behavior, fecal and hair cortisol
Marinelli et al. [54]	AAA, AAT	Resident, Visitation	Hospitals, clinics or rehabilitation centers, schools, nursing homes	Adults, children	18	Behavior, handler questionnaire
King et al. [55]	AAT	Visitation	Hospital	Adults, children	21	Salivary cortisol, behavior, handler questionnaire
Glenk et al. [56]	AAT	Visitation	In-patient mental healthcare	Adults	21	Salivary cortisol
Barstad [57] **^1^**	AAA, AAT	Visitation	Nursing homes	Adults	13	Behavior, handler questionnaire, cognitive test
Glenk et al. [58]	AAT	Visitation	In-patient substance abuse treatment	Adults	5	Salivary cortisol, behavior
Ng et al. [59]	AAA	Visitation	University	Adults	15	Salivary cortisol, behavior
Palestrini et al. [60]	AAT	Visitation	Pediatric hospital	Children	1	Heart rate, behavior

**^1^** Master thesis.

**Table 2:** AAI session characteristics including duration, organization of recipients (single or group sessions), between session intervals and significant findings.

References	Duration	Single/Group	Intervals	Significant Findings
Haubenhofer and Kirchengast [52,53]	1–8 h	- **^2^**	Differed from 9–50 sessions/3 months	↑ Salivary cortisol: on working days, during short sessions with high intensity, high frequency of sessions
Piva et al. [28]	20 min	Group	3–4 sessions/week	↓ Stereotypic autogrooming; ↑ play behavior, socialization; ↓ hair cortisol
Marinelli et al. [54]	10–105 min	Single, group	Daily	↑ Stress-related behavior if recipients were children < 12 years; increase in the frequency of sessions and number of recipients across 3 years
King et al. [55]	2 h	Single	Biweekly	No effect of a short time-out session; ↑ salivary cortisol after 60 min; ↑ behavioral signs of stress in dogs < 6 years and/or < 2 years of AAI experience
Glenk et al. [56]	50–60 min	Group	Weekly	No difference between working and resting days; ↓ salivary cortisol in therapy dogs off-lead
Barstad [57] **^1^**	30 min	Group	Biweekly	No differences in cognitive task performance before 12 weeks of AAIs and pre-post session; no changes in behavioral variables; ↑ responsiveness to commands; ↓ focus on handler
Glenk et al. [58]	55–60 min	Group	Weekly	↓ Salivary cortisol in session 4 and 5; no changes in behavior
Ng et al. [59]	60 min	Group	- **^2^**	No difference between working and resting days; ↑ salivary cortisol in novel environment
Palestrini et al. [60]	20 min	Single	- **^2^**	No changes in heart rate or behavior across 20 sessions

**^1^** Master's thesis; **^2^** Information not available.
